# A Programmable, DNA-Exclusively-Guided Argonaute DNase and Its Higher Cleavage Specificity Achieved by 5′-Hydroxylated Guide

**DOI:** 10.3390/biom12101340

**Published:** 2022-09-21

**Authors:** Shichao Sun, Dejin Xu, Lin Zhu, Bei Hu, Zhen Huang

**Affiliations:** 1 Key Laboratory of Bio-Resource and Eco-Environment of Ministry of Education, College of Life Sciences, Sichuan University, Chengdu 610064, China; 2 Study on the Structure-Specific Small Molecule Drug in Sichuan Province College Key Laboratory, Department of Pharmacy, Chengdu Medical College, Chengdu 610500, China; 3 SeNA Research Institute and Szostak-CDHT Large Nucleic Acids Institute, Chengdu 610041, China

**Keywords:** Argonaute, mesophilic bacterium, *Clostridium disporicum*, DNase, specificity

## Abstract

Argonaute proteins exist widely in eukaryotes and prokaryotes, and they are of great potential for molecular cloning, nucleic acid detection, DNA assembly, and gene editing. However, their overall properties are not satisfactory and hinder their broad applications. Herein, we investigated a prokaryotic Argonaute nuclease from a mesophilic bacterium *Clostridium disporicum* (CdAgo) and explored its overall properties, especially with 5′-hydroxylated (5′-OH) guides. We found that CdAgo can exclusively use single-stranded DNA (ssDNA) as guide to cleave ssDNA and plasmid targets. Further, we found the length of the efficient guide is narrower for the 5′-OH guide (17–20 nt) than for the 5′-phosphorylated guide (5′-P, 14–21 nt). Furthermore, we discovered that the 5′-OH guides can generally offer stronger mismatch discrimination than the 5′-P ones. The 5′-OH guides offer the narrower length range, higher mismatch discrimination and more accurate cleavage than the 5′-P guides. Therefore, 5′-OH-guide-directed CdAgo has great potential in biological and biomedical applications.

## 1. Introduction

Argonaute (Ago) proteins exist in all three domains of life. Eukaryotic Argonaute proteins (eAgos) are a core of eukaryotic RNA interference (RNAi) machinery [1,2], which use RNA guides to recognize and cleave complementary RNA targets. Prokaryotic Argonautes (pAgos) are found in bacteria and archaea [3,4,5,6], such as TtAgo from *Thermus thermophilus* [7], PfAgo from *Pyrococcus furiosus* [8], and MjAgo from *Methanocaldococcus jannaschii* [9]. These pAgos prefer to cleave DNA in vitro and protect cells from foreign genetic elements in vivo [4]. Many new functions of pAgos have also been found recently, such as regulating DNA replication [10], providing robust antiviral protection via activating membrane effectors [11], and triggering NAD(P)^+^ depletion and cell death upon RNA-guided detection of invading DNA [12]. Based on the programmable cleavage activity, pAgos have successfully been used in nucleic acid detection [13,14], molecular cloning [15], and DNA assembly [16,17]. In addition, compared with the widely used gene-editing system (CRISPR-Cas), pAgos do not require the protospacer adjacent motif (PAM) sequences [18,19], thereby having great potential as next-generation tools.

Based on the domain architecture, pAgos can be divided into three large clades, including short pAgos, long-A, and long-B pAgos [20]. The majority of long pAgos have domain architectures similar to eAgos, including N, PAZ, MID, and PIWI domains, while short pAgos lack N and PAZ domains. Most long-A pAgos share a conserved tetrad of amino acid residues (DEDX, where X is D, H, or K) in the PIWI domain, which are involved in catalysis [21]. 5′ and 3′ ends of the guide in pAgo-guide complexes are anchored in the MID and PAZ domains, respectively [22]. The guide 5′-region binds first with the target, followed by its binding with the guide 3′-region, forming the ternary complex [23]. Though generally offering sequence specificity (mismatch discrimination), guides of most mesophilic pAgos (such as the CpAgo [24], IbAgo [24], CbAgo [25,26], LrAgo [26], SeAgo [27], and KmAgo [28,29]) are more discriminating in their 3′-regions. When loaded with the guides, pAgos can cleave complementary targets at the site opposite to the position between the 10th and 11th nt (from 5′-end) of the guide [30,31,32] or between the 11th and 12th nt [24,26,27,28,29].

Though sharing high structural homology with eAgos, most characterized pAgos have different functions and preferences for guides and targets. Most identified pAgos can utilize a much broader spectrum of guides and substrates (DNA and RNA) than eAgos. For instance, MpAgo (from *Marinitoga piezophila*) [32] can use RNA guides to cleave ssDNA and ssRNA, TtAgo (from *Thermus thermophilus*) [22] can use DNA guides to cleave ssDNA and ssRNA, CbAgo can use DNA or RNA guides to cleave ssDNA [26], and KmAgo can use DNA or RNA guides to cleave ssDNA and ssRNA [28,29]. Recognizing more than one type of guide and/or target may lead to increase in off-target products. In addition, the minimal lengths of the guides vary from 9 to 14 nt, such as 9 nt for TtAgo [23] and KmAgo [29], 12 nt for CpAgo and IbAgo [24], and 14 nt for CbAgo [25,26]. The guide lengths can affect the nucleic acid cleavages: short guides [28,29,33] and long guides [29,34] can cause the off-target risk. Further, mismatches between guides and targets are tolerated by pAgos [26,29], thus reducing cleavage specificity. Furthermore, the literature has indicated that DNA-DNA duplexes are the most discriminative among DNA-DNA, RNA-RNA, and DNA-RNA duplexes [33,35]. Therefore, we hypothesized that a pAgo DNase with the DNA guide specificity is the most desired. On the basis of the reported sequence and analysis, we considered Argonaute nuclease from a mesophilic bacterium *Clostridium disporicum* (CdAgo) [20], matching our expectation.

Herein, we report our CdAgo characterization and discriminative strategy for high cleavage specificity. Namely, to increase the mismatch discrimination, we investigated the 5′-OH DNA guides potentially with high paring differentiation since the 5′-P end interacts with CdAgo canonically [36]. We thus hypothesized that the 5′-OH end might reduce binding affinity and therefore improve mismatch discrimination. In our research, we compared CdAgo with many other pAgos, and we found that CdAgo has five major advantages: (1) the high specificity in exclusively selecting the DNA guides and DNA targets; (2) the high mismatch discrimination in the presence of 5′-OH gDNA; (3) high cleavage site specificity in the presence of 5′-OH gDNA as some pAgos cleaving targets at multiple sites [26,27,28]; (4) the narrow guide-length range of 5′-OH gDNA; and (5) the satisfactory catalytic activity, considering that several pAgos have quite low activities [37]. In conclusion, we consider CdAgo as a versatile DNA cleavage tool with great potential for many biological and biomedical applications.

## 2. Materials and Methods

### 2.1. Protein Expression and Purification

The gene of CdAgo (WP_055276084.1; *Clostridium disporicum*) was codon-optimized for expression in *Escherichia coli*, synthesized, and cloned into pET28a expression vector by Sangon Biotech (Shanghai, China). The mutant (inactive CdAgo with D538A and D608A) was obtained by site-directed mutagenesis kit (Tsingke, Beijing, China). The wild-type (WT) and the double-mutant CdAgo (D538A and D608A, named CdAgo_DM) were expressed in *Escherichia coli* BL21 (DE3) (TransGen, Beijing, China), which were grown in Luria–Bertani (LB) medium containing 50 μg·mL^−1^ kanamycin for 4 h at 37 °C and induced with 0.2 mM Isopropyl-D-thiogalatopyranoside (IPTG) for 20 h at 18 °C. The cells were harvested by centrifugation and disrupted by a high-pressure homogenizer (Scientz, Ningbo, China) at 800 bars in buffer A (20 mM Tris-HCl, pH 7.5, 500 mM NaCl, 10 mM imidazole, 1.4 mM β-Mercaptoethanol, 1 mM PMSF). Following a high-speed centrifugation, the supernatant was loaded onto a nickel column (HisTrap FF, GE Healthcare, Chicago, IL, USA) and washed with buffer A containing supplementary 60 mM imidazole. Then, the bound proteins were eluted with buffer A containing supplementary 300 mM imidazole. The elution was diluted by adding buffer B (20 mM Tris-HCl, pH 7.5, 150 mM NaCl, 10 mM imidazole, 1 mM DTT) and loaded on a heparin column (HiTrap Heparin HP, GE Healthcare) to remove nucleic acids and purify the proteins. A further gel-filtration purification was performed on a size exclusion column (Superdex 200 Increase 10/300 GL, GE Healthcare) and eluted with Buffer C (20 mM HEPES, 0.5 M NaCl, 1 mM DTT, 5% glycerol). The purified CdAgo proteins were diluted to a final concentration of 8 μM with Buffer C and were flash-frozen in liquid nitrogen and stored at −80 °C. The protein purity was assessed by SDS-PAGE with Coomassie staining.

### 2.2. Single-Stranded Nucleic Acid Cleavage Assays

All guide and target oligonucleotides (sequences shown in Table S1) were synthesized by Sangon (Shanghai, China). All cleavage assays were performed at the 4:2:1 pAgo: guide: target molar ratio at 37 °C unless otherwise indicated. CdAgo (800 nM) was mixed with the ssDNA or ssRNA guides (400 nM) in reaction buffer containing 10 mM HEPES-NaOH (pH 7.0), 100 mM NaCl, 5 mM MnCl_2_, and 5% glycerol, and the reaction mixture was incubated at 37 °C for 10 min for guide loading. Then, 5′-FAM-labeled ssDNA or RNA targets (200 nM) were added, followed by an incubation at 37 °C for 2 h unless otherwise indicated. The reaction was terminated by mixing the samples with equal volumes of stop solution (8 M urea, 20 mM EDTA, 0.005% (*w*/*v*) Bromophenol Blue, 0.005% (*w*/*v*) Xylene Cyanol, 30% (*v*/*v*) glycerol). After a heating for 5 min at 95 °C, the cleavage reactions were analyzed on a 20% urea-denaturing polyacrylamide gel electrophoresis (urea-PAGE), visualized with Gel Doc™ XR+ (Bio-Rad, California, USA), quantified by the ImageJ software, and graphed by Prism 8 (GraphPad) software. To explore the pH impact on CdAgo, the CdAgo was mixed with ssDNA guides in reaction buffer with different pH, followed by an incubation at the same pH. To test whether the pre-treatment (preloading) of CdAgo first with guides can stabilize CdAgo, purified nucleic-acid-free CdAgo was preloaded with guide DNA (gDNA) and incubated at different temperatures for 30 min, followed by cleavage activity assays on ssDNA target.

### 2.3. Double-Stranded DNA Cleavage Assays

Four 5′-P-ssDNA guides (18 nt) were synthesized, including two NC guides (N1 and N2, non-complementary to the target pclone007 plasmid) and two guides targeting the plasmid at the opposite site (F1 and R1 guides). Moreover, five guide sets (each set contained F and R guides) with various GC-contents (24, 29, 40, 51, and 60%) targeting the plasmid were designed and synthesized (Table S2). The cleavage analysis was performed to verify whether the target GC contents could influence the CdAgo cleavage process. The linearized plasmid was generated by a treatment of the supercoiled plasmid with Sca I for 10 min at 37 °C. In experiment, CdAgo was loaded with F or R guide separately in two pre-treatments (buffer conditions: 10 mM HEPES-NaOH (pH 7.0), 100 mM NaCl, 5 mM MnCl_2_, 5% glycerol) for 30 min at 55 °C. Then, the two pre-treatments were mixed, followed by addition of 130 ng target plasmid (supercoiled or linearized ones). After the mixture was incubated at 37 °C or 65 °C for 2 h, the CdAgo reaction was quenched by treating with Proteinase K at 25 °C for 20 min and analyzed on 1.2% agarose gel.

### 2.4. Guide-Independent Activity Assays

Chopping reactions (guide-independent) were performed with CdAgo (8 pmol) and DNAs (130 ng for each reaction, including dsDNA PCR product, linearized plasmid DNA, supercoiled plasmid DNA, *E. coli* BL21 genomic DNA, or human genomic DNA) under the reaction conditions (10 mM HEPES-NaOH (pH 7.0), 100 mM NaCl, 5 mM MnCl_2_, 5% glycerol). The reactions were incubated at 37 °C for 2 h and terminated by Proteinase K, followed by analysis on 1.2% agarose gel.

## 3. Results

### 3.1. CdAgo Cleaves ssDNA Targets with DNA Guides at 37 °C

**Figure 1:**
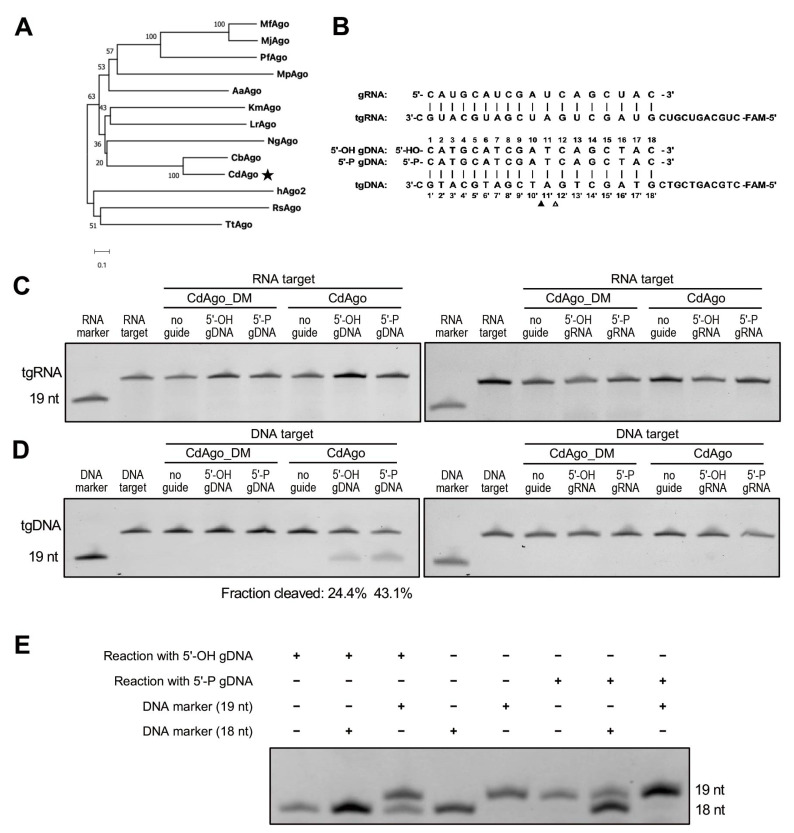
CdAgo exhibits exclusively DNA-guided DNA cleavage activity at 37 °C. (**A**) Schematic phylogenetic tree of CdAgo based on amino acid sequences. The asterisk indicates the pAgo studied in this work. Bootstrap values are indicated near the nodes. (**B**) Sequences of synthetic guides and targets used in the cleavage assays in (**C**,**D**). Nucleotides of the pairing guides and target are numbered. The filled and unfilled triangles indicate the cleavage site with 5′-P and 5′-OH guide DNA, respectively. (**C**) Cleavage activity assays with the FAM-labeled ssRNA target. The reaction time was 1 h. (**D**) Cleavage activity assays with the FAM-labeled ssDNA target. gRNA, guide RNA; tgRNA, target RNA; gDNA, guide DNA; tgDNA, target DNA. The reaction time was 1 h. (**E**) PAGE analysis of CdAgo cleavage site.

Recent research on comparison of pAgo sequences and activities revealed that pAgos are strikingly more diverse than eAgos. To identify a highly specific pAgo that is selective for a specific type of guide and target and has a narrow range of guide length and a strong potential to discriminate mismatches between guide and target, we chose the protein sequence of CbAgo (WP_045143632.1) as the query for its high activity at moderate temperature and used the web interface of the BLASTp program to search for mesophilic pAgos. We sought out a particular Argonaute from a mesophilic anaerobic bacillus named *Clostridium disporicum*, which had 55.88% sequence identity, 100% query cover, and a 0.0 E-value with CbAgo (Figure 1A). CdAgo is a long-A pAgo and has conserved catalytic tetrad residues (D538, E574, D608, and D724) in the PIWI domain (Figure S1A), and its molecular weight is 86 kDa. To study the biochemical properties of CdAgo, we expressed and purified CdAgo and CdAgo_DM (with D538A and D608A double mutations in the catalytic tetrad) in *E. coli* using a T7-based pET expression system, and the purification of CdAgo was analyzed with SDS-PAGE after gel filtration (Figure S1B–D). To study its preference for substrate and guide types, CdAgo was investigated in vitro by using RNA and DNA guides (with 5′-OH or 5′-P) with the synthetically FAM-labeled oligonucleotides (RNA and DNA) as the substrates (Figure 1B). We found that neither RNA nor DNA guides directed CdAgo to cleave the RNA substrate (Figure 1C). The DNA guide directed CdAgo to exclusively cleave the DNA substrate (Figure 1D), while the sRNA guide did not. These finding indicated that CdAgo only cleaves DNA targets by using DNA guides, which is consistent with two recent reports [17,38]. Further, no DNA cleavage was observed when CdAgo_DM was used, suggesting that the integrity of the DEDD catalytic tetrad in the PIWI domain was essential for the cleavage activity (Figure 1C,D). Furthermore, we observed that the cleaved products with 5′-OH and 5′-P gDNA were 18 and 19 nt, respectively (Figure 1E).

### 3.2. CdAgo Assembles Guides and Functions Efficiently under a Wide Range of Reaction Conditions

**Figure 2:**
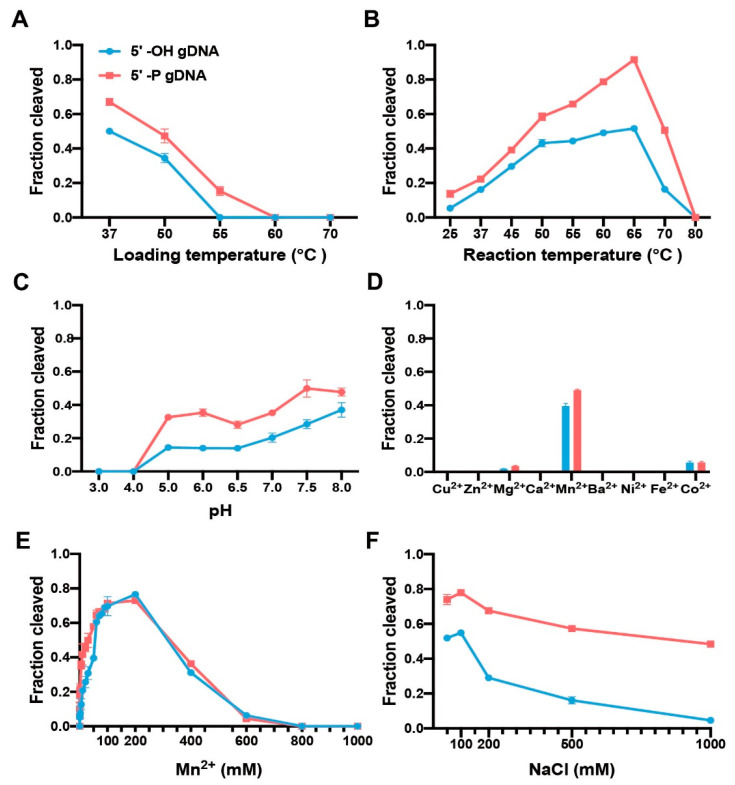
Impact of reaction conditions on CdAgo catalysis. (**A**) The loading temperature impact (guide-CdAgo assembly). (**B**) The reaction temperature impact. The reaction time was 1 h. (**C**) The pH impact. The reaction time was 1 h. (**D**) The divalent cation impact. The cation concentration was 5 mM. (**E**) The Mn^2+^ concentration impact. (**F**) The NaCl concentration impact (see also Figure S2). Means and standard deviations from three independent experiments are shown in each panel.

To explore the loading temperature for guide-CdAgo assembly, we performed the CdAgo loading at different temperatures (37, 50, 55, 60, or 70 °C) for 10 min. Subsequently, these CdAgo assemblies were used to cleave the DNA target at 37 °C. We found that the optimal loading temperature was 37 °C. Furthermore, when the loading temperature was increased to 55 °C, CdAgo was active in the presence of the 5′-P gDNA while inactive with the 5′-OH guide (Figure 2A and Figure S2A).

To analyze temperature sensitivity of CdAgo, we performed cleavage reactions at various temperatures from 25 to 80 °C (Figure 2B and Figure S2B). The results indicated that the 5′-P DNA guide offered higher cleavage efficiency than the corresponding 5′-OH guide, and reaction efficiencies increased in both cases with temperature rising from 25 to 65 °C and decreasing rapidly from 70 and 80 °C, suggesting a broad temperature range (25 to 70 °C) of CdAgo.

To identify the optimal pH for CdAgo activity, the cleavage reaction was investigated at different pH (3–14) conditions. We found that with both 5′-OH and 5′-P guides, CdAgo was inactive at a pH of 4 or lower, while its activity increased by elevating pH from 5 to 8 (Figure 2C and Figure S2C). Interestingly, it was still active at pH 13 (Figure S2C).

Since divalent cations are indispensable for Ago activity [39], we tested the divalent cation preference of CdAgo. The results indicated that Mn^2+^ assisted CdAgo with the highest activity, while Mg^2+^ and Co^2+^ offered much less assistance, and other divalent cations (Cu^2+^, Zn^2+^, Ca^2+^, Ba^2+^, Ni^2+^, and Fe^2+^) did not support cleavage at all (Figure 2D and Figure S2D). Further, titration of Mn^2+^ ions showed that CdAgo achieved the highest activity at 200 mM Mn^2+^ concentration, while it was inactive above 600 mM Mn^2+^ (Figure 2E and Figure S2E). Since high ionic strength can inhibit pAgo activity [8,24], we explored this by varying the concentrations of NaCl. The results showed that CdAgo was active in a wide range of NaCl concentration (from 50 to 500 mM, Figure 2F and Figure S2F), with the highest activity observed at 100 mM NaCl.

### 3.3. CdAgo Shows Narrower Guide Length Range with 5′-OH Guides Than 5′-P Guides

**Figure 3:**
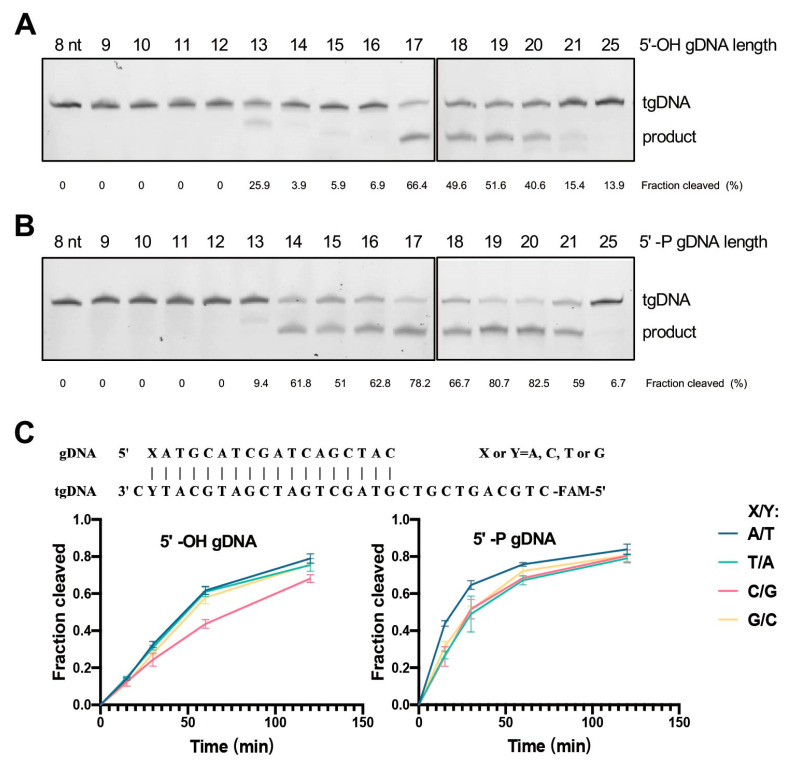
Effects of guide length and guide 5′-terminus on CdAgo activity. (**A**) PAGE analysis of cleavage with the 5′-OH guides in various lengths. (**B**) PAGE analysis of cleavage with the 5′-P guides in various lengths. (**C**) Kinetic analysis of the CdAgo activities with the 5′-OH or 5′-P guides containing four different 5′-terminal nucleotides (see also Figure S3). Means and standard deviations from three independent experiments are shown.

As Ago proteins bind nucleic acid guides of varying sequence length, which can influence the cleavage activities, we wanted to figure out the best lengths for CdAgo activation. Therefore, we performed the reactions using the identical target and a set of 5′-OH and 5′-P gDNAs with the lengths varied from 8 to 25 nt, sharing an identical 5′-region (Table S1). We found that CdAgo was mainly active in the presence of 17–20 nt long guides (5′-OH or 5′-P) (Figure 3A,B). The 5′-P guides with 14–16 or 21 nt length could assist CdAgo to cleave the target with comparable activity, while the 5′-OH guides could not. Interestingly, the shorter guides with 13 nt (both 5′-OH and 5′-P) could still assist CdAgo to cleave the target but at a different site (Figure 3A,B).

### 3.4. CdAgo Has No Preference on 5′-End Nucleotides

In order to investigate CdAgo preference for the 5′-terminal guide nucleotide, we designed the guides (both 5′-OH and 5′-P) with all four different 5′-terminal nucleotides (Table S1). We found that CdAgo proteins loaded with the guides containing a 5′-A, C, G, or T terminus offered nearly the same cleavage efficiency (Figure 3C and Figure S3).

### 3.5. CdAgo Performs More Sensitive to Mismatches with 5′-OH Guides than 5′-P Guides

**Figure 4:**
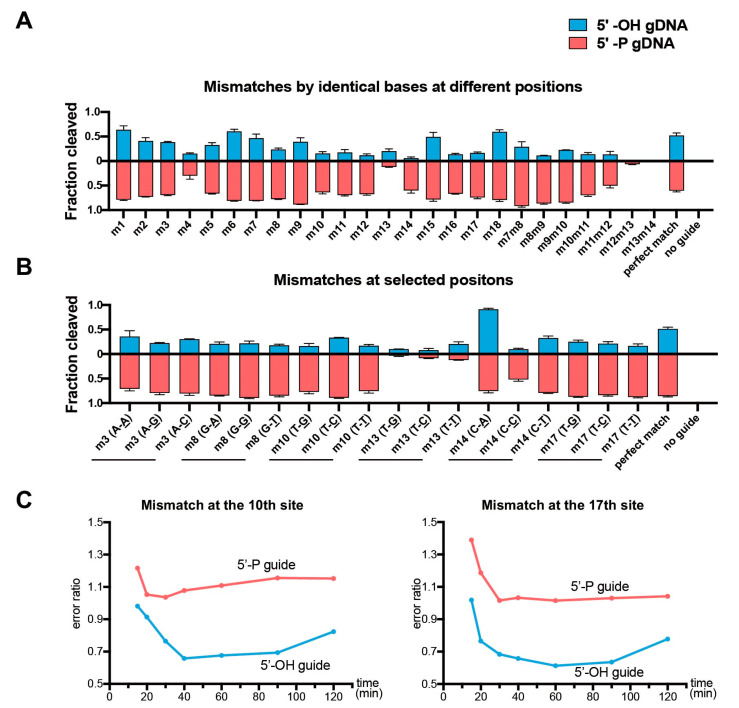
Effects of the guide-target mismatches on the cleavage activity of CdAgo. Quantitative analysis of the cleavage fraction with the 5′-OH or 5′-P guides containing mismatches. (**A**) The catalytical effects of the identical base-paring mismatches at all positions. (**B**) The catalytic effects of selected mismatches at various positions. (**C**) Error ratio of CdAgo cleavage. The error ratio is defined as the ratio of cleavage product yield for the mismatched versus matching guide at the same reaction time point (Yield(mismatched)/Yield(matched)). The corresponding urea-PAGE analyses are shown in Figure S4. Means and standard deviations from three independent experiments are shown in (**A**,**B**).

In order to explore the mismatch discrimination of CdAgo, we designed a set of DNA guides (5′-OH and 5′-P) forming single or double mismatches with the target at various positions (Table S1). In the presence of the 5′-OH gDNAs, the cleavage efficiency of CdAgo on complexes with single mismatches (otherwise identical base paring between the target and guide) was lower than for the perfectly matching guide except for mismatches at positions 1, 6, and 18 (Figure 4A and Figure S4A). However, in the presence of the 5′-P gDNAs, the cleavage efficiency of CdAgo on substrates with single mismatches were higher than on the perfectly matching substrate except for the mismatches at positions 4 and 13 (Figure 4A and Figure S4A). Further, we investigated double mismatches, and we found that the double mismatches around position 13 (m12m13 and m13m14) caused CdAgo discrimination, while other double mismatches (such as m7m8, m8m9, m9m10, m10m11, and m11m12) did not. Furthermore, we explored various selected positions with all three types of mismatches between the guides and targets. The results showed that most of the mismatches on the same position yielded similar CdAgo activities (Figure 4B and Figure S4B). Moreover, cleavage kinetics with mismatched gDNAs indicated that the 5′-OH guides decrease the CdAgo error ratio nearly over the entire course of the reaction (Figure 4C and Figure S4C). In general, CdAgo was more sensitive to all mismatch types at most positions in the presence of the 5′-OH gDNA than the 5′-P gDNA.

### 3.6. CdAgo Cleaves Plasmid DNA in the Guide-Dependent Manner

**Figure 5:**
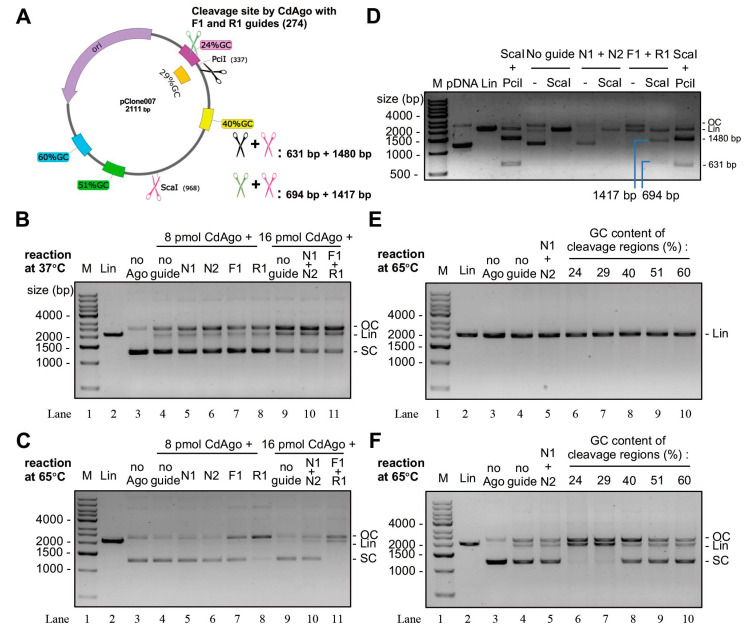
Guided cleavage of supercoiled plasmid DNA by CdAgo. (**A**) The target regions of the plasmid in the cleavage reactions in (**B**–**D**). (**B**) The CdAgo cleavage of supercoiled plasmid at 37 °C. (**C**) The CdAgo cleavage of supercoiled plasmid at 65 °C. (**D**) The double digestions by ScaI and CdAgo in the presence of F1 and R1. pDNA, supercoiled plasmid DNA. (**E**) CdAgo reactions with linearized plasmid DNA and gDNAs directed against different target sequences (see panel A) with varying GC content. (**F**) As panel E, but using supercoiled plasmid DNA. M, DNA markers; SC, supercoiled plasmid DNA; OC, open circular plasmid; Lin, linearized plasmid DNA.

To explore whether CdAgo can cleave double-stranded DNA (dsDNA) in the presence of DNA guides, we performed the cleavage reactions using the 5′-P DNA guides (Table S2) targeting supercoiled or linearized plasmid DNA (Figure 5A). We found that at 37 °C, the cleaved products were formed in the absence of guide DNA (Lane 4) or in the presence of N1 or N2 guides (Lane 5 or 6; non-complementary to the plasmid) when compared with the negative control (Lane 3) (Figure 5B). This is regarded as the guide-independent activity (chopping). However, at 65 °C, this chopping activity was hardly observed. Further, in the presence of DNA guides (F1 or R1, complementary to the plasmid), more cleaved products were formed in the presence of these specific guides (Lanes 7, 8, and 11 in Figure 5C) at 65 °C compared with their corresponding controls in the absence of the specific guides (Lanes 4, 5, 6, 9, and 10). However, there was no obvious difference with and without the specific guides at 37 °C (Figure 5B). To investigate whether the cleavages with F1 and/or R1 were performed in the guide-dependent manner (canonical cleavage activity), we further digested the CdAgo-linearized plasmid using the restriction endonuclease (ScaI, Figure 5A). We found that two cleavage fragments were formed (Figure 5D), as predicated in Figure 5A (694 and 1417 bp), similar to the products cleaved by PciI and ScaI (Figure 5D) whose lengths were 631 and 1480 bp (Figure 5A). These results indicated that CdAgo is able to cleave supercoiled plasmid in the guide-dependent manner, while it is unable cleave linearized plasmid DNA (Figure 5E).

To investigate whether the GC content could affect canonical CdAgo cleavage activity, five guide sets (each set containing the complementary F and R guides; Table S2) with various GC content were designed and synthesized (Figure 5A). The results demonstrated that CdAgo can cleave the supercoiled plasmid when the GC content was low (such as 24% and 29%) in the target region, while higher GC contents (40, 51, and 60%) significantly reduced cleavage activity (Figure 5F). These results suggested that the GC content plays a key role for cleavage of supercoiled plasmid DNA.

### 3.7. CdAgo Cleaves Double-Stranded DNA in the Guide-Independent Manner (Chopping)

**Figure 6:**
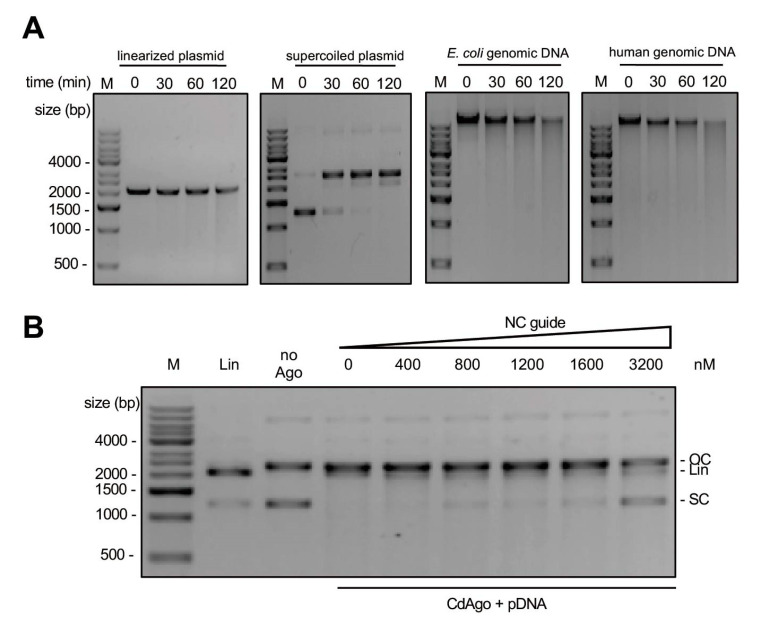
The CdAgo chopping activity. (**A**) Chopping of various DNA substrates with CdAgo at 37 °C. (**B**) Inhibition of the CdAgo chopping activity by increasing concentrations of NC guide (non-complementary guide DNA) at 37 °C.

Almost all DNA-guided pAgos have chopping activity [9,25,26,27,28,29,40], cleaving target DNA in a guide-independent manner and generating the digested DNAs. To investigate the chopping activity of CdAgo, we performed a set of cleavage reactions without DNA guides (Figure 6A). The results showed that in the absence of guides, CdAgo chopped these double-stranded DNA substrates, including linearized plasmid, supercoiled plasmid, *E. coli* genomic DNA, and human genomic DNA. Further, on account of the competition between guide-dependent and guide-independent activity, we hypothesized that guide strands occupying the active site will inhibit the chopping activity. Such inhibition of chopping activity was indeed observed when reactions contained increasing concentrations of non-complementary gDNA (Figure 6B).

## 4. Discussion

Several mesophilic pAgos have been reported recently and allowed exploration of their mechanism with the goal to utilize them in biomedicine and biotechnology. However, their overall properties, including the high specificity in selecting the guides and targets, cleaving-site specificity, guide-length range, catalytic activity, and mismatch discrimination, are not yet satisfactory. To face these challenges, we identified and characterized the pAgo DNase from the mesophilic bacterium *Clostridium disporicum* (CdAgo) and discovered that it exclusively uses DNA guides to cleave DNA targets efficiently (Figure 1). In addition, we explored a strategy with high specificity by using 5′-OH guides to minimize off-target effects (Figure 3 and Figure 4).

Since a pAgo using dual types of guides (DNA and RNA) can cause higher off-target effects than a pAgo using a single type (DNA or RNA), the latter is preferred over the former; likewise, DNA guides are preferred over RNA guides, as the former are more selective [33,35,41]. Further, the guide length can affect the cleavage site and manner (guide-dependent or chopping activity) [28,29]. Herein, we categorized the DNAs (< 17, 17–20, > 20 nt) as short, appropriate-length, and long DNA guides, respectively. When the length range is 17–20 nt, most pAgos perform canonical cleavage exclusively (at a single site between 10′ and 11′ nucleotide), while many pAgos perform uncanonical cleavages when the guide length is shorter than 17 nt or longer than 20 nt. The short guides increase the probability of directing pAgos to multiple complementary sites, which increases the potential to cause off-target effects. For instance, short guides (< 17 nt) directing pAgo to the human genome (3.16 billion bp) have more than one fully complementary target site, at least theoretically. In addition, the short and long guides can usually shift the cleavage site [29,34] because the guide–pAgo affinity may be reduced in the presence of long guide [9], which could release the guide from the guide–pAgo complex, resulting in the forming of guide–target duplex. This duplex can induce the chopping activity at non-canonical sites in the desired region [9].

Furthermore, as indicated by previous studies on the pAgo mismatch discrimination, an imperfectly paired target can induce dissociation of guide or guide/target duplex from ternary Ago complex [42,43]. As it was also reported that pAgo can recognize the guide 5′-P [36], we hypothesized that the guide 5′-OH had weaker interaction with pAgo, probably resulting in better mismatch discrimination, which might explain the better selectivity with the 5′-OH guide (Figure 4).

Although CdAgo has chopping activity similar to other DNA-guided pAgos [9,25,26,27,28,29,40,41], which can hinder their applications, two strategies can be explored to suppress the chopping activity. As shown in Figure 5 and Figure 6 and some previous studies [26,28,29], one strategy is to increase the reaction temperature in order to prevent the guide-free pAgos from cleaving dsDNA nonspecifically. The other one is to increase guide quantity in order to occupy the pAgo active site for inhibiting the chopping activity, as depicted in Figure 6B. Obviously, the combination of these two strategies can minimize the chopping activity.

Among all identified pAgos from mesophilic bacteria or archaea, most of them recognize DNA guides and DNA targets non-exclusively. These pAgos also recognize RNA guides and/or targets, such as CpAgo, CbAgo, KmAgo, and RsuAgo (Table S3), increasing the risk of off-target effects. Further, when using 5′-P gDNA, CdAgo is one of the pAgos with relative a narrow range of guide length (Table S3). When using 5′-OH gDNA, the range is narrower (Table S3), offering more CdAgo cleavage accuracy. Furthermore, even though most of these pAgos can use 5′-OH DNA as a guide, this guide reduces the cleavage activity (as in the case of PbAgo and BlAgo) and causes multiple cleavage sites (such as LrAgo, SeAgo, and KmAgo) (Table S3). Fortunately, CdAgo can efficiently cleave targets at a specific site when programmed with 5′-OH guides. Moreover, 5′-OH gDNA can enhance mismatch discrimination, while 5′-P gDNA cannot (Table S4). In general, compared to other characterized mesophilic pAgos, CdAgo has many potentials in biotechnological and biomedical applications.

## 5. Conclusions

In conclusion, we investigated and characterized a pAgo DNase from a mesophilic bacterium, *Clostridium disporicum* (CdAgo). We have found that CdAgo can exclusively use ssDNA guides to cleave ssDNA as well as double-stranded, supercoiled plasmid DNA targets. Further, we discovered that CdAgo uses 5′-OH DNA guides with a length of 17–20 nt while utilizing 14–21 nt long 5′-P DNA guides. Furthermore, we illustrated that the 5-OH guides can generally offer higher mismatch-discrimination than 5′-P ones. Collectively, the 5-OH guides have higher length and sequence specificity than the 5′-P ones. Clearly, the 5-OH-guide-directed CdAgo has great potentials in biological and biomedical applications.

## Data Availability

Not applicable.

## Supplementary Materials

The following supporting information can be downloaded at: https://www.mdpi.com/article/10.3390/biom12101340/s1, Figure S1. CdAgo Purification; Figure S2. Urea-PAGE analysis of reactions with 5-OH gDNA or 5′-P gDNA as guides in different reaction conditions; Figure S3. Urea-PAGE analysis of reaction by using different 5-end nucleotides of gDNAs; Figure S4. Gel analysis of effects of guide-target mismatches on the cleavage activity of CdAgo; Figure S5. CdAgo stabilization with gDNA; Table S1. Sequences of oligonucleotides used in the ssDNA cleavage assays; Table S2. Sequences of oligonucleotides used in the plasmid DNA cleavage assays; Table S3. Enzymatic characters of different mesophilic pAgos. Table S4. Positions sensitive to mismatch in guide-dependent cleavage by different mesophilic pAgos. Reference [44] is cited in the supplementary materials.
