# Supplementary material for: A Programmable, DNA-Exclusively-Guided Argonaute DNase and Its Higher Cleavage Specificity Achieved by 5′-Hydroxylated Guide
Source: Biomolecules. 2022 Sep 21;12(10):1340. doi: 10.3390/biom12101340 (PMC9599953; doi:10.3390/biom12101340)
Supplement: Supplementary file 1 [file biomolecules-12-01340-s001.zip › biomolecules-1878813-supplementary.pdf]

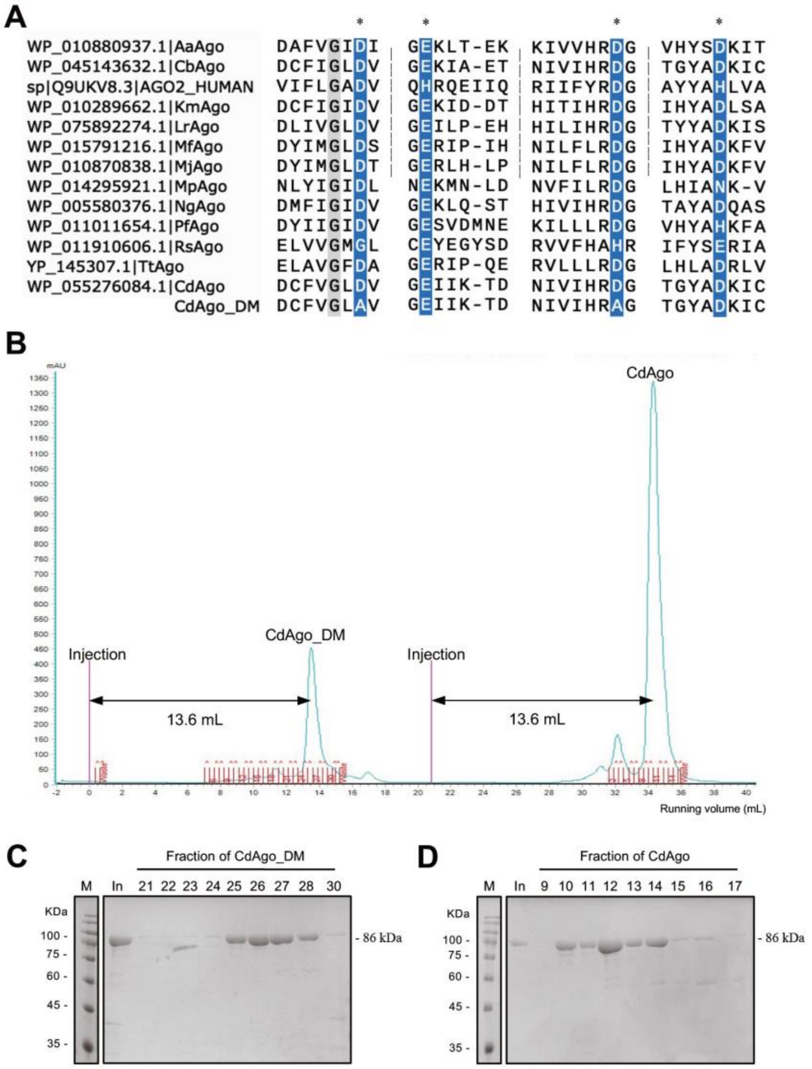

Figure S1. CdAgo Purification. **(A)** Alignment of CdAgo catalytic DEDD tetrad with several other biochemically or structurally characterized Ago proteins. CdAgo_DM is an inactive mutant of CdAgo with two substituted amino acid residues within the catalytic tetrad. The conserved amino acid residues in the PIWI domain of pAgos are marked by asterisks. **(B)** Gel-filtration purification of CdAgo_DM and CdAgo. **(C)** and **(D)** SDS-PAGE analysis of purified CdAgo_DM and CdAgo, respectively.

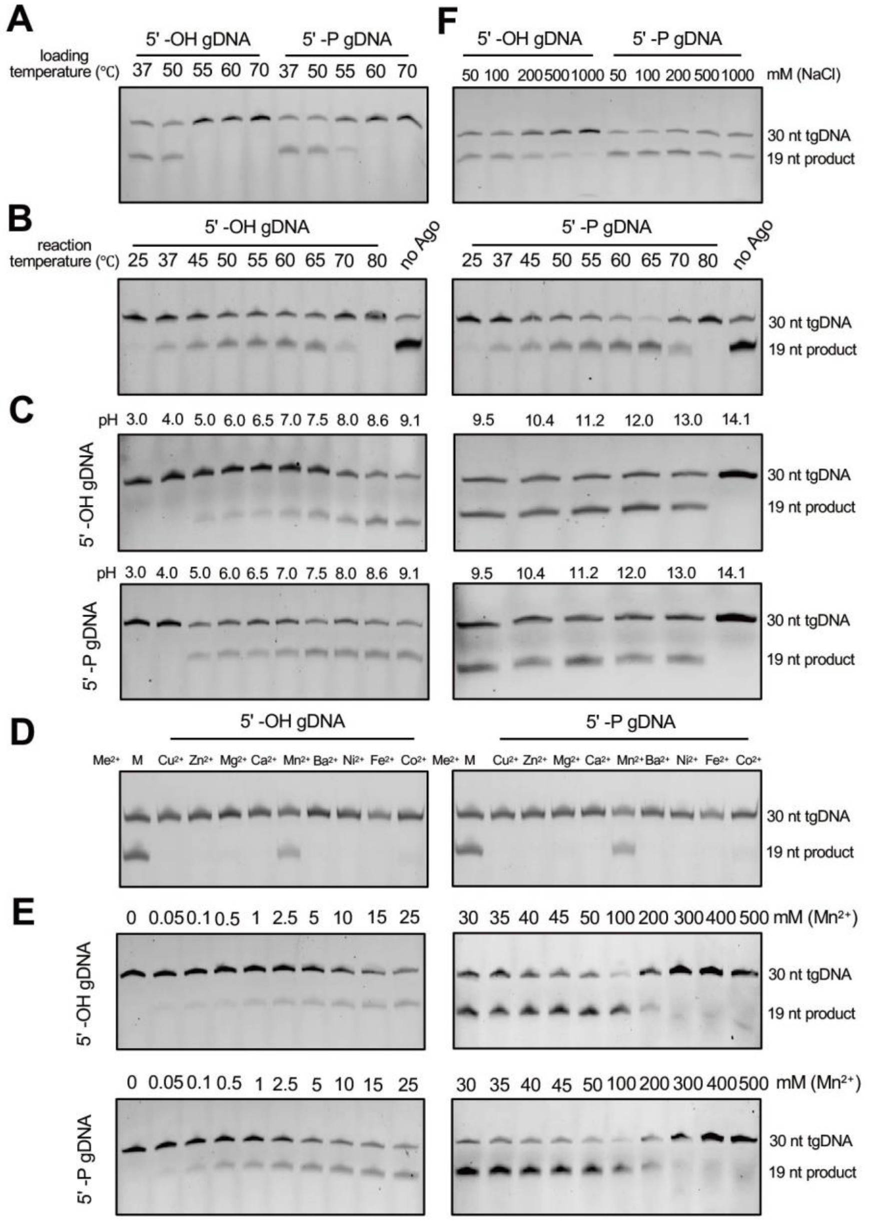

Figure S2. Urea-PAGE analysis of reactions with 5'-OH gDNA or 5'-P gDNA as guides in different reaction conditions. **(A)** Effects of loading temperature. **(B)** Effects of reaction temperature. **(C)** Effects of pH. **(D)** Effects of type of divalent cation. **(E)** Effects of Mn^{2+} concentration. **(F)** Effects of NaCl concentration. Note: when the pH was above 9.1, Mn^{2+} formed $MnOH_2$ precipitation.

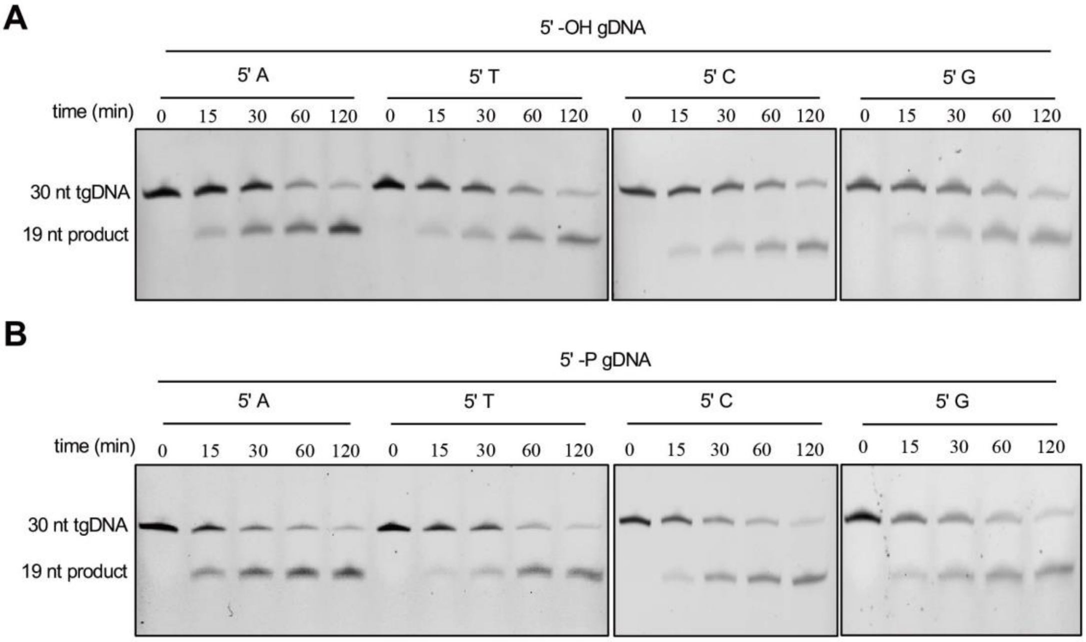

Figure S3. Urea-PAGE analysis of reaction by using different 5'-end nucleotides of gDNAs. **(A)** 5'-OH gDNA with different 5'-end nucleotides. **(B)** 5'-P gDNA with different 5'-end nucleotides. Quantification analyses of the cleavage efficiencies are shown in Figure 3C.

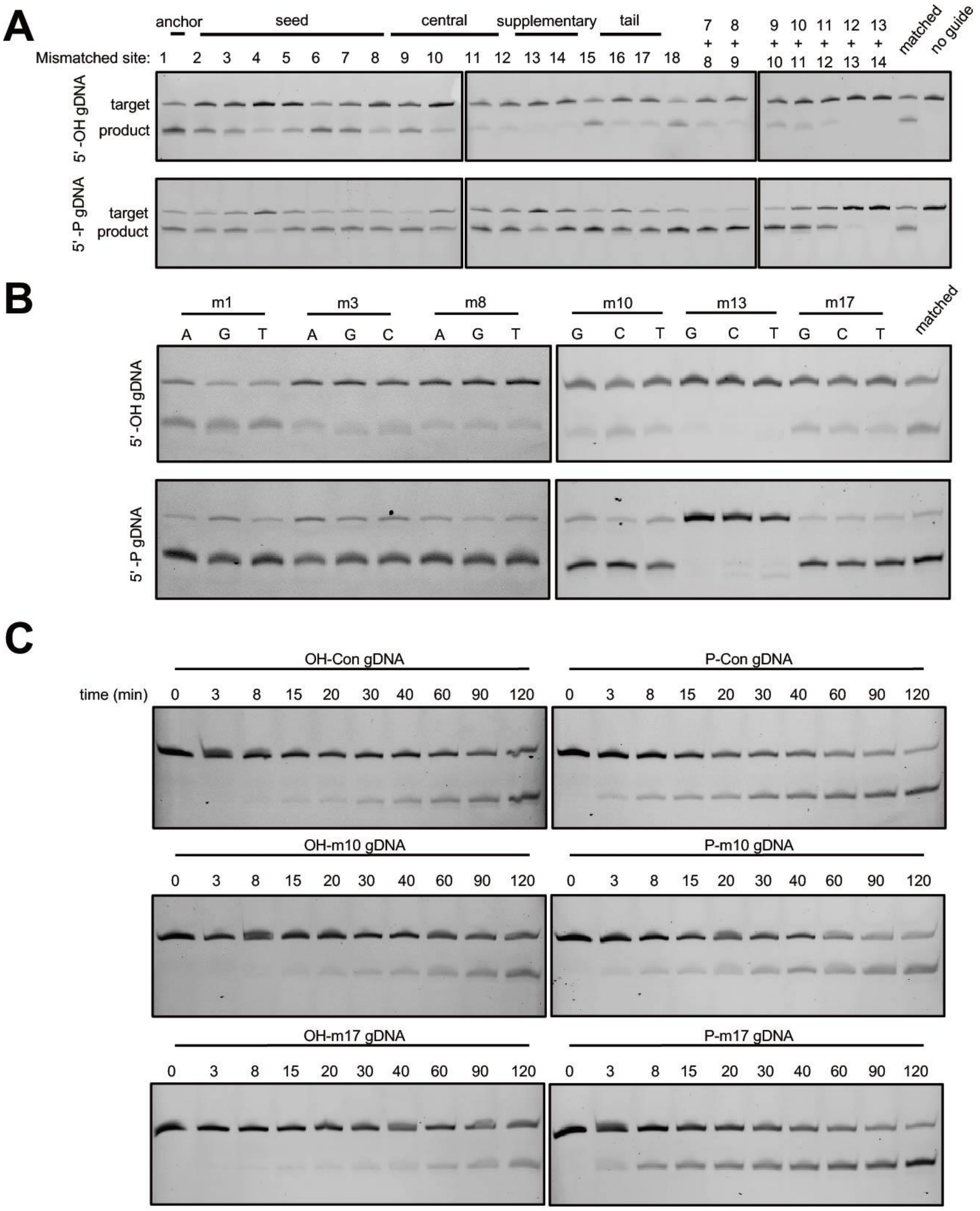

Figure S4. Gel analysis of effects of guide-target mismatches on the cleavage activity of CdAgo. **(A)** Identical base-paring mismatches at all positions. **(B)** Selected mismatches at various positions. **(C)** Error ratio of CdAgo in reaction process. The error ratio is defined as the ratio of cleavage product yield for the mismatched versus matching guide at the same reaction time point (Yield(mismatched)/Yield(matched)).

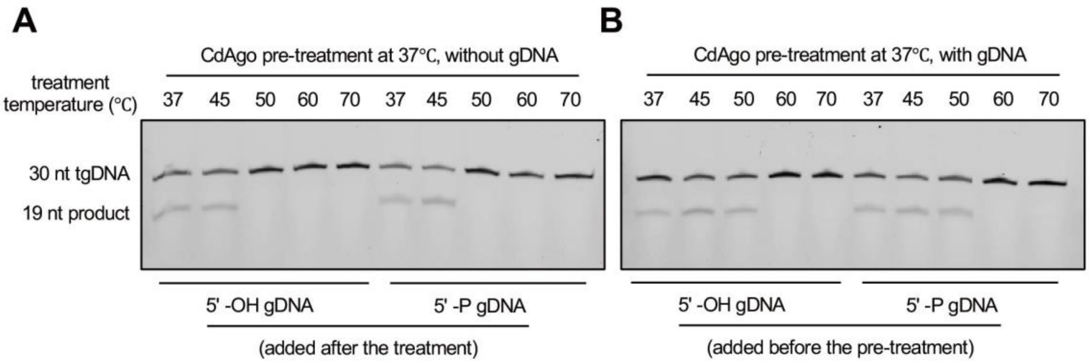

Figure S5. CdAgo stabilization with gDNA. **(A)** CdAgo pre-treatment (37°C for 10 min) without gDNA was followed by the treatments (37, 45, 50, 60, or 70°C for 30 min) and gDNA loading (37°C for 10 min), then the reaction substrate was added into system, followed by 1 h reaction and gel analysis. **(B)** CdAgo pre-treatment (37°C for 10 min) with gDNA was followed by the treatments (37, 45, 50, 60, or 70°C for 30 min), then the reaction substrate was added into system, followed by 1 h reaction and gel analysis.

Table S1. Sequences of oligonucleotides used in the ssDNA cleavage assays.

Oligonucleotide name	Sequence (5' -3')	Description
30 nt DNA target	FAM-d (CTGCAGTCGTCGTAGCTGATCGATGCATGC)	Target
30 nt RNA target	FAM-r (CTGCAGTCGTCGTAGCTGATCGATGCATGC)	
19 nt DNA product	FAM-d(CTGCAGTCGTCGTAGCTGA)	Marker
18 nt DNA product	FAM-d(CTGCAGTCGTCGTAGCTG)	
17 nt DNA product	FAM-d(CTGCAGTCGTCGTAGCT)	
19 nt RNA product	FAM-r(CUGCAGUCGUCGUAGCUGA)	
C-gRNA	r(CAUGCAUCGAUCAGCUAC)	For studying on preferences of guide and target types
G-tgRNA	FAM-r(CUGCAGUCGUCGUAGCUGAUCGAUGCAUGC)	
A-gDNA	d(**A**ATGCATCGATCAGCTAC)	For studying on preferences of 5'-end nucleotide of DNA guides
T-gDNA	d(**T**ATGCATCGATCAGCTAC)	
C-gDNA	d(**C**ATGCATCGATCAGCTAC)	
G-gDNA	d(**G**ATGCATCGATCAGCTAC)	
T-tgDNA	FAM-d(CTGCAGTCGTCGTAGCTGATCGATGCAT**T**C)	
A-tgDNA	FAM-d(CTGCAGTCGTCGTAGCTGATCGATGCAT**A**C)	
G-tgDNA	FAM-d(CTGCAGTCGTCGTAGCTGATCGATGCAT**G**C)	
C-tgDNA	FAM-d(CTGCAGTCGTCGTAGCTGATCGATGCAT**C**C)	
8 nt gDNA	d(CATGCATC)	For studying on preferences of the length of DNA guides
9 nt gDNA	d(CATGCATCG)	
10 nt gDNA	d(CATGCATCGA)	
11 nt gDNA	d(CATGCATCGAT)	
12 nt gDNA	d(CATGCATCGATC)	
13 nt gDNA	d(CATGCATCGATCA)	
14 nt gDNA	d(CATGCATCGATCAG)	
15 nt gDNA	d(CATGCATCGATCAGC)	
16 nt gDNA	d(CATGCATCGATCAGCT)	
17 nt gDNA	d(CATGCATCGATCAGCTA)	
18 nt gDNA	d(CATGCATCGATCAGCTAC)	
19 nt gDNA	d(CATGCATCGATCAGCTACG)	
20 nt gDNA	d(CATGCATCGATCAGCTACGA)	
21 nt gDNA	d(CATGCATCGATCAGCTACGAC)	
25 nt gDNA	d(CATGCATCGATCAGCTACGACGACT)	
gDNA_mm1	d(GATGCATCGATCAGCTAC)	For studying on mismatched guide cleavage activity
gDNA_mm2	d(CTTGCATCGATCAGCTAC)	
gDNA_mm3	d(CAAGCATCGATCAGCTAC)	
gDNA_mm4	d(CATCCATCGATCAGCTAC)	
gDNA_mm5	d(CATGGATCGATCAGCTAC)	
gDNA_mm6	d(CATGCTTCGATCAGCTAC)	
gDNA_mm7	d(CATGCAACGATCAGCTAC)	
gDNA_mm8	d(CATGCATGGATCAGCTAC)	
gDNA_mm9	d(CATGCATCCATCAGCTAC)	
gDNA_mm10	d(CATGCATCGTTCAGCTAC)	
gDNA_mm11	d(CATGCATCGAACAGCTAC)	
gDNA_mm12	d(CATGCATCGATGAGCTAC)	
gDNA_mm13	d(CATGCATCGATCTGCTAC)	

gDNA_mm14	d(CATGCATCGATCACCTAC)	
gDNA_mm15	d(CATGCATCGATCAGGTAC)	
gDNA_mm16	d(CATGCATCGATCAGCAAC)	
gDNA_mm17	d(CATGCATCGATCAGCTTC)	
gDNA_mm18	d(CATGCATCGATCAGCTAG)	
gDNA_m7m8	d(CATGCAAGGATCAGCTAC)	
gDNA_m8m9	d(CATGCATGCATCAGCTAC)	
gDNA_m9m10	d(CATGCATCCTTCAGCTAC)	
gDNA_m10m11	d(CATGCATCGTACAGCTAC)	
gDNA_m11m12	d(d(CATGCATCGAAGAGCTAC)	
gDNA_m12m13	d(CATGCATCGATGTGCTAC)	
gDNA_m13m14	d(CATGCATCGATCTCCTAC)	
gDNA_mm1 (A)	d(AATGCATCGATCAGCTAC)	
gDNA_mm1 (T)	d(TATGCATCGATCAGCTAC)	
gDNA_mm3 (C)	d(CACGCATCGATCAGCTAC)	
gDNA_mm3 (G)	d(CAGGCATCGATCAGCTAC)	
gDNA_mm8 (A)	d(CATGCATAGATCAGCTAC)	
gDNA_mm8 (T)	d(CATGCATTGATCAGCTAC)	
gDNA_mm10 (C)	d(CATGCATCGCTCAGCTAC)	
gDNA_mm10 (G)	d(CATGCATCGGTCAGCTAC)	
gDNA_mm13 (C)	d(CATGCATCGATCCGCTAC)	
gDNA_mm13 (G)	d(CATGCATCGATCGGCTAC)	
gDNA_mm17 (C)	d(CATGCATCGATCAGCTCC)	
gDNA_mm17 (G)	d(CATGCATCGATCAGCTGC)	

Note: The 5'-end nucleotides of gDNA and corresponding 3'-end nucleotides of tgDNA are marked by red bold; mismatch sites between gDNA and tgDNA are marked by red color.

Table S2. Sequences of oligonucleotides used in the plasmid DNA cleavage assays.

oligonucleotide name	sequence (5'-3')	GC content of target region (%)
N1	CTATCCTTCCAAGGAAGA	45
N2	CTTCTTCCTTGGAAGGAT	45
F1	TTTTCATATAAAGGTGAG	24
R1	AACTCACCTTTATATGAA	24
F2	TCAAAAAGGATCTTCACC	29
R2	TAGGTGAAGATCCTTTTT	29
F3	AATGAAATAAGATCACTA	40
R3	GGTAGTGATCTTATTTCA	40
F4	TGAAGCCATACCAAACGA	51
R4	CGTCGTTTGGTATGGCTT	51
F5	GCCGGTGAGCGTGGGTCT	60
R5	CGAGACCCACGCTCACCG	60

Table S3. Enzymatic characters of different mesophilic pAgos.

pAgo	cleavage activity with various guide/target type								range of effective guide DNA[1]		cleavage site[2]		reference
	DNA-targeted				RNA-targeted								
	DNA-guided		RNA-guided		DNA-guided		RNA-guided		5'-OH	5'-P	5'-OH	5'-P	
	5'-OH	5'-P	5'-OH	5'-P	5'-OH	5'-P	5'-OH	5'-P					
CdAgo	+[3]	+	-	-	-	-	-	-	17–20	14–21	11'-12'	10'-11'	this work
PbAgo	+	+	-	-	-	-	-	-	/	14–21	11'-12'	10'-11'	[1]
BlAgo	+	+	-	-	-	-	-	-	/	14–35	11'-12'	10'-11'	[1]
IbAgo	+	+	-	-	-	-	-	-	/	15–30	11'-12'	10'-11'	[2]
LrAgo	+	+	/	-	-	-	/	-	/	16–20[4]	11'-12'<br>12'-13'	10'-11'	[3]
SeAgo	+	+	/	-	/	-	/	-	/	/	10'-11'<br>11'-12'	10'-11'	[4]
CbcAgo	-[5]	+	/	/	/[5]	/	/	/	/	11–19[6]	-	10'-11'	[5]
CpAgo	+	+	-	-	+	+	-	-	/	14–30	11'-12'	10'-11'	[2]
CbAgo	+	+	/	+	-	+	/	-	/	14–22[6]	10'-11'	10'-11'	[3,6]
RsuAgo	+	+	-	+	-	+	-	-	/	15–25	10'-11'	10'-11'	[7]
KmAgo	+	+	-	+	+	+	-	+	/	9–40	9'-10'<br>11'-12'	10'-11'	[8,9]

Note: [1]DNA-targeted cleavage with 5′-OH or 5′-P guide; [2]Only DNA-targeted activities are shown, and the numbering of cleavage site is consistent with it in Figure 1B; [3]plus sign indicates there is obvious product detected; [4]the longest guide they tested is effective; [5]minus sign indicates there is no obvious product detected; [6]slash sign indicates the assay was not performed.

Table S4 Positions sensitive to mismatch in guide-dependent cleavage by different mesophilic pAgos.

5'-end type of guide	pAgo	position sensitive to mismatch[1]					reference
		5'-nucleotide (1)	seed region (2-8)	central region (9-12)	supplementary region (13-15)	tail region (16-18)	
5′-OH	CdAgo	-[2]	2-5	8-12	13-14	16-17	this work
5′-P	CdAgo	-	4	-	13	-	this work
	CpAgo	-	-	12	13-15	-	[2]
	IbAgo	-	-	-	14-15	16	[2]
	CbAgo	-	5-6	10, 12	13-15	-	[3]
	LrAgo	-	-	10-12	13-15, 17	-	[3]
	SeAgo	-	-	10-12	13-15	16, 18	[4]
	PbAgo	-	-	11-12	-	-	[1]
	BlAgo	-	-	10-12	-	-	[1]
	KmAgo	-	4-5	9-12	13-15	16	[8,9][3]
	RsuAgo	-	3	11	15	17	[7]

[1]It is defined as sensitive when mismatch induced yield decrease is over 20%; [2]minus sign indicates none of positions is sensitive to mismatch; [3]sensitive positions shown were found exclusively from the study [8].

References

1. Dong, H.; Huang, F.; Guo, X.; Xu, X.; Liu, Q.; Li, X.; Feng, Y. Characterization of Argonaute nucleases from mesophilic bacteria Paenibacillus borealis and Brevibacillus laterosporus. *Bioresources and Bioprocessing* **2021**, *8*, 133. https://doi.org/10.1186/s40643-021-00478-z.
2. Cao, Y.; Sun, W.; Wang, J.; Sheng, G.; Xiang, G.; Zhang, T.; Shi, W.; Li, C.; Wang, Y.; Zhao, F.; et al. Argonaute proteins from human gastrointestinal bacteria catalyze DNA-guided cleavage of single- and double-stranded DNA at 37 degrees C. *Cell Discov* **2019**, *5*, 38. https://doi.org/10.1038/s41421-019-0105-y.
3. Kuzmenko, A.; Yudin, D.; Ryazansky, S.; Kulbachinskiy, A.; Aravin, A.A. Programmable DNA cleavage by Ago nucleases from mesophilic bacteria Clostridium butyricum and Limnothrix rosea. *Nucleic Acids Res* **2019**, *47*, 5822-5836. https://doi.org/10.1093/nar/gkz379.
4. Olina, A.; Kuzmenko, A.; Ninova, M.; Aravin, A.A.; Kulbachinskiy, A.; Esyunina, D. Genome-wide DNA sampling by Ago nuclease from the cyanobacterium Synechococcus elongatus. *RNA Biol* **2020**, *17*, 677-688. https://doi.org/10.1080/15476286.2020.1724716.
5. Garcia-Quintans, N.; Bowden, L.; Berenguer, J.; Mencia, M. DNA interference by a mesophilic Argonaute protein, CbcAgo. *F1000Res* **2019**, *8*, 321. https://doi.org/10.12688/f1000research.18445.2.
6. Hegge, J.W.; Swarts, D.C.; Chandradoss, S.D.; Cui, T.J.; Kneppers, J.; Jinek, M.; Joo, C.; van der Oost, J. DNA-guided DNA cleavage at moderate temperatures by Clostridium butyricum Argonaute. *Nucleic Acids Res* **2019**, *47*, 5809-5821. https://doi.org/10.1093/nar/gkz306.
7. Jiang, X.; Liu, Y.; Liu, Q.; Ma, L. Characterization of a Programmable Argonaute Nuclease from the Mesophilic Bacterium Rummeliibacillus suwonensis. *Biomolecules* **2022**, *12*, 355. https://doi.org/10.3390/biom12030355.
8. Kropocheva, E.; Kuzmenko, A.; Aravin, A.A.; Esyunina, D.; Kulbachinskiy, A. A programmable pAgo nuclease with universal guide and target specificity from the mesophilic bacterium Kurthia massiliensis. *Nucleic Acids Res* **2021**, *49*, 4054-4065. https://doi.org/10.1093/nar/gkab182.
9. Liu, Y.; Li, W.; Jiang, X.; Wang, Y.; Zhang, Z.; Liu, Q.; He, R.; Chen, Q.; Yang, J.; Wang, L.; et al. A programmable omnipotent Argonaute nuclease from mesophilic bacteria Kurthia massiliensis. *Nucleic Acids Res* **2021**, *49*, 1597-1608. https://doi.org/10.1093/nar/gkaa1278.
